# Changes in total body fat and body mass index among children with juvenile dermatomyositis treated with high-dose glucocorticoids

**DOI:** 10.1186/s12969-021-00622-1

**Published:** 2021-08-10

**Authors:** Amer Khojah, Victoria Liu, Gabrielle Morgan, Richard M. Shore, Lauren M. Pachman

**Affiliations:** 1 Division of Pediatric Rheumatology, Department of Pediatric, Ann & Robert H. Lurie Children’s Hospital of Chicago, Chicago, IL 60611 USA; 2 Division of Allergy & Immunology, Ann & Robert H. Lurie Children’s Hospital of Chicago, Chicago, IL USA; 3 Cure JM Center of Excellence, Stanley Manne Research Center, Chicago, IL USA; 4 Department of Medical Imaging, Ann & Robert H. Lurie Children’s Hospital of Chicago, Chicago, IL USA

**Keywords:** Juvenile dermatomyositis, Pediatric obesity, Glucocorticoids, DXA scan, And fat distribution

## Abstract

**Objective:**

High-dose glucocorticoids (GC) remain the primary therapy to induce remission in Juvenile Dermatomyositis (JDM). Studies of the natural history of GC associated weight gain in children are very limited, especially in the JDM population. This study aims to measure BMI changes in a cohort of JDM subjects over 60 months and to examine the changes in body composition by DXA.

**Methods:**

We included all subjects with JDM who had 5 years of follow-up data and multiple DXA studies. BMI and total body fat (TBF) percentiles were calculated based on the CDC published percentile charts. To study the natural history of weight gain and TBF, we assessed the data at four-time points (T0 = baseline, T1 > 1.5 years, T2 = 1.51–3.49 years, T3 = 3.5–5 years).

**Results:**

68 subjects (78% female, 70% white) were included in this retrospective study. Paired T-test showed a significant increase in the mean BMI percentile by 17.5 points (*P* = 0.004) after the initiation of medical treatment, followed by a gradual decrease over the study period. However, the TBF percentile did not change over the study period. TBF in the last visit (T3) had a strong correlation with the T1 BMI, and T1 TBF percentile (correlation coefficients 0.63, 0.56 *P* < 0.001, 0.002 respectively). Also, there was a positive correlation (correlation coefficients 0.39, *P* = 0.002) between the TBF percentile and muscle DAS but not the skin DAS.

**Conclusions:**

Although the BMI percentile decreased throughout the study, the TBF percentile remained high until the end of the study (60 months). This finding raises the concern that some of the reduction in the BMI percentile could reflect a drop in the lean body mass from muscle wasting rather than actual fat loss.

**Supplementary Information:**

The online version contains supplementary material available at 10.1186/s12969-021-00622-1.

## Introduction

Juvenile Dermatomyositis (JDM) is a multisystem pediatric disease characterized by chronic inflammation of muscle and skin [1]. Despite the recent advances in the treatment of JDM, high dose corticosteroid remains the primary therapy to induce remission [2]. Typically, JDM patients need either intravenous or oral corticosteroid therapy for around 2 years [1]. Although previous studies showed that untreated JDM subjects have lower weight and height than age and gender-matched controls, [3] weight gain and cushingoid features are among the most common side effects observed in children after glucocorticoid initiation in various clinical trials [4]. Excessive weight gain has a negative impact on the affected child’s physical and psychological well-being [5]. Youth obesity is associated with an increased risk of hypertension, type 2 diabetes mellitus, and hyperlipidemia, which are significant risk factors for future cardiovascular diseases [6]. Cardiovascular diseases are an important cause of mortality and morbidity in patients with inflammatory myopathy [7].

Besides corticosteroid therapy, there are other possible mechanisms of obesity in JDM subjects, such as; the lack of physical activity due to muscle weakness and metabolic changes from chronic inflammation [8, 9]. Long term studies of the natural history of corticosteroid associated weight gain in children are very limited, especially in the JDM population. Loss of muscle mass due to muscle inflammation and muscle ischemia may complicate the assessment of obesity and mask total gain in adipose tissue.

Dual-energy X-ray absorptiometry (DXA) has been used to measure various body composition, including body fat with high-precision and relatively low X-ray exposure [10, 11]. JDM patients typically undergo routine DXA to measure the bone density due to their increased risk of pathological fractures from active inflammation, decrease mobility, and the chronic use of steroids [12]. The same DXA scan was used in this study to assess body composition, including total body fat, without additional financial burden to the patients or extra radiation. This study aims to measure body mass index (BMI) changes in a cohort of JDM subjects over 60 months duration and examine the body composition changes (fat vs. lean body mass) by DXA.

## Methods

This was a retrospective chart review study conducted at The CureJM Center of Excellence in Juvenile Myositis Research and Care, Ann & Robert H. Lurie Children’s Hospital between 2000 and 2017 (IRB# 2012–14,858). We included all JDM subjects who met Bohan and Peter criteria for definite or probable JDM diagnosis and had a minimum of 5 years of follow-up data with multiple DXA and BMI assessments during the study period. Subjects with overlap syndrome were excluded from the analysis. A GE-LUNAR iDXA bone densitometer was used to perform the DXA. Encore 16 software was used to analyzed DXA results and assess fat distribution among the various body part. The TBF (total body fat) percentile was calculated based on the Centers for Disease Control and Prevention (CDC) published TBF percentile charts from National Health and Nutrition Examination Survey (NHANES) [13].

The BMI percentile was calculated based on CDC published charts (https://www.cdc.gov/growthcharts/clinical_charts.htm). Overweight was defined as a BMI at or above the 85th percentile and below the 95th percentile for age and gender-matched children. Obesity was defined as a BMI at or above the 95th percentile for age and gender-matched children. To study the natural history of weight gain and body fat changes, we assessed the patients’ data at four-time point based on the duration of time between the date of first medication use and date of an assessment (T0 = baseline, T1 > 1.5 years, T2 = 1.51–3.49 years, T3 = 3.5–5 years). Of note, if there is more than one assessment for a specific time point, we chose the closest to the optimal time point. For example, if a subject had DXA at 1.5 years and 2.5 years we only included the 2.5 years for T2 time point. We also evaluated disease activity markers on presentation such as skin, muscle weakness, and total Disease Activity Score (DAS) [14]. We defined disease course groups as following: 1-Monophasic: achieved remission without subsequent flares requiring reinitiation of treatment. 2- Polyphasic: remission achieved within any length of time with at least one flare requiring treatment adjustment 3-Chronic: failed to achieve remission and remain on treatment.

All statistical analyses were done IBM SPSS Statistics 26® software. The paired T-test was used to compare the baseline BMI and TBF data and the subsequent time points. Person correlation analysis was used to assess the association between TBF percentile at the last visit of the study (3.5–5 years after medication onset) and disease activity markers and initial BMI. A *P*-value less than 0.05 was considered significant. The figures were generated using Graphpad Prism 8 software.

## Results

**Fig. 1:**
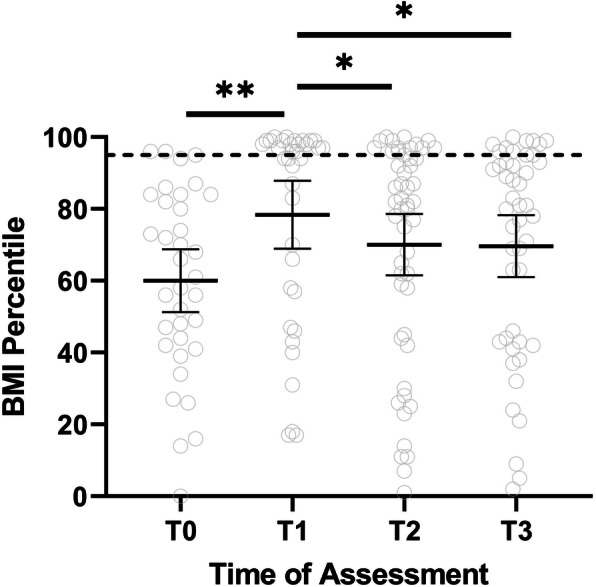
Changes of BMI percentile in JDM patients. T0 represented baseline date (before initiation of treatment), note data available for around 40% of the study subject. Other time point are T1 > 1.5 years, T2 = 1.51–3.49 years, and T3 = 3.5–5 years. The dotted line represents the 95th percentile; children with BMI above the 95th percentile meet the CDC definition of childhood obesity. Paired T-test showed a significant increase in the mean BMI percentile by 17.5 points (*P =* 0.004) after the initiation of medical treatment. BMI percentile decrease over the study period. Of note, * means *P* value between 0.05–0.01 and ** means *P* value between 0.01–0.001

**Fig. 2:**
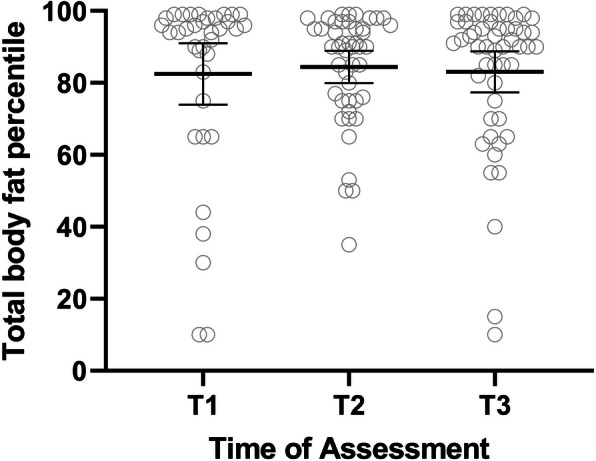
Changes of Total body fat (TBF) percentile in JDM patients. T1 > 1.5 years, T2 = 1.51–3.49 years, and T3 = 3.5–5 years. Paired T-test did not show a significant change in the TBF over the study period

Sixty-eight children with definite JDM were included in the study. The demographic data and disease subgroups by Myositis Specific Antibodies are available in Table 1. The mean age at enrolment was 6.97 years (+/− 3.4 SD). The mean duration of untreated disease was 6.7 months (+/− 8.4 SD). Disease course categories and metabolic complications were included in Table 2. All study subjects received corticosteroid therapy, and many had multiple other immunosuppressive therapies during the study period (Table 1). The mean duration of oral corticosteroid in the study was 2.9 years (+/− 3.4 SD) and the mean number of IV steroid pulses for the subjects who received them was 29 (+/− 22 SD). BMI before the start of treatment revealed that only 16% of JDM patients were either overweight or obese. Of note, 53% of study subjects had BMI assessment before starting medications because the rest of the patients has received therapy before their initial assessment in our center. The mean time since the start of therapy for study visits was 0.96 +/− 0.4 years, 2.56+/− 0.4 years, and 4.33 +/− 0.4 years for T1, T2, and T3, respectively. The majority of the children,for whom baseline BMI data were available, had a significant weight increase after treatment; 64% were either in the overweight or obese category using the CDC definitions, and 30% of them were more than 98th percentile BMI for their age (Supplement Fig. 1). The baseline BMI Paired T-test showed a significant increase in the mean BMI percentile by 17.5 points (*P* = 0.004) after the initiation of medical treatment (T1 time point) (Fig. 1). The average weight gain from baseline at the T1 time point was around 7.5 kg (mean weight at baseline 26.59 kg, mean weight at T1 34.13 kg *P* < 0.001). Unfortunately, most JDM subjects did not have DXA at baseline; therefore, assessing the change in the TBF from pretreatment was not done. Although the BMI percentile significantly decreased over the study period (Fig. 1), the TBF percentile did not change over the study period (Fig. 2, Supplement Fig. 2). TBF in the last visit (T3) had a strong correlation with the T1 BMI, and T1 TBF percentile (correlation coefficients 0.63, 0.56 *P <* 0.001, 0.002 respectively) (Supplement Fig. 3). Interestingly, there is a positive correlation between the TBF percentile at T3 and muscle DAS (correlation coefficients 0.49, *P* = 0.001) (Supplement Fig. 4) but not with the skin DAS (correlation coefficients − 0.08, *P* = 0.61). Also, there was a negative correlation between CMAS (Childhood Myositis Assessment Scale) and TBF percentile at T3 (correlation coefficients − 0.42, *P* = 0.027). Of note, there was no significant correlation between the duration of oral corticosteroid therapy and BMI or TBF percentile.

**Table 1:** Demographic and disease characteristics of the study cohort

	Frequency (n)	Percentage
Sample size	68	
Gender		
Female	54	79.4%
Male	14	20.6%
Race/Ethnicity		
White	48	70.6%
Hispanic	10	14.7%
African American	6	8.8%
Others	4	5.9%
Age		
Less than 5 years	26	38.2%
More than 5 years	42	61.8%
Treatment status at 1st visit		
Untreated	36	52.9%
Treated	32	47.1%
Myositis specific antibodies		
P155/140	29	42.6%
MJ	5	7.2%
Mi2	5	7.2%
MDA5	2	2.9%
Others or multiple MSAs	1	1.5%
Negative	26	38.2%
Treatment		
Oral steroid	68	100%
Intravenous steroid	60	88.2%
Methotrexate	66	97.1%
Intravenous immunoglobulin	14	20.6%
Hydroxychloroquine	28	41.2%
Cyclosporin	19	27.9%
Mycophenolate	43	63.2%

**Table 2:** Disease Course and complication of the study cohort

	Frequency (n)	Percentage
Sample size	68	
Disease course		
Monophasic	43	63.2%
Polyphasic	12	17.6%
Chronic	12	17.6%
Lipodystrophy (ever)		
Present	20	29.4%
Absent	48	70.6%
Calcification (ever)		
Present	8	11.8%
Absent	60	88.2%
History of fracture		
Before the JDM diagnosis	3	4.4%
After the JDM diagnosis	13	19.4%
Absent	51	76.1%
Dyslipidemia		
Acceptable Triglyceride level	26	48.1%
Borderline Triglyceride level	11	20.4%
High Triglyceride level	17	31.5%
Acceptable Total Cholesterol level	32	59.3%
Borderline Total Cholesterol level	15	27.8%
High Total Cholesterol level	7	13.0%
Acceptable LDL Cholesterol level	37	72.5%
Borderline LDL Cholesterol level	7	13.7%
High LDL Cholesterol level	7	13.7%
Acceptable HDL Cholesterol level	32	61.5%
Borderline HDL Cholesterol level	8	15.4%
low HDL Cholesterol level	12	23.1%

## Discussion

Although most JDM patients had a normal BMI before starting medication, 70% met the CDC definition of overweight or obese in the first data point of the study (0.95 +/− 0.4 years after treatment initiation). This rapid increase in the patient’s weight is likely due to corticosteroid therapy, physical inactivity, and changes in adipokine levels due to their inflammatory status [4, 8, 15]. Although the BMI percentile improves over the study period, it did not reach pretreatment level even 4.5 years after the start of therapy. This highlights the importance of preventing excessive weight gain as soon as a patient gets started on chronic corticosteroid with early institution of exercise and healthy eating habits. Of note, the percentage of children with calcifications was 11%, which is less than the current national level 13.3% [1]. Unfortunately, in our study, we could not assess the extent of their physical activity and adipokine due to the study’s retrospective nature. Although we could not measure the exact accumulative dose of corticosteroid, most patients in our study had prolonged oral steroid courses with a mean duration of oral steroid around 3 years and multiple IV steroid courses. The typical treatment plan of JDM patient in our center involves a minimum of 3 days of IV methylprednisolone pulse (30 mg/kg with maximum dose 1000 mg) on admission followed by weekly IV methylprednisolone pulses with small doses of oral steroids (typically 0.5 mg/kg/day of prednisolone), which were weaned over time base the disease’s activity. Of note, seven subjects (five of them were managed in other centers initially) did not receive IV steroid therapy in our cohort. These subjects had lower DAS (6.6 vs 12 *P* < 0.001), lower Neopterin (10.2 vs 19 *P* = 0.08) and higher CMAS (43.7 vs 30.4 *P* = 0.03). Even though steroid-sparing agents such as methotrexate, mycophenolate, and IVIG are typically started early on, our JDM patients generally are treated with glucocorticoids for at least 1–2 years. This significant corticosteroid exposure can lead to an increased risk of side effects such as weight gain, glaucoma, cataracts, and vertebral fracture. Our patients usually get DXA annually to monitor bone density. The same DXA scan was used in this study to assess body composition, including total body fat, without additional financial burden to the patients or extra radiation. Assessing body composition by DXA revealed a higher percentage of total body fat than the age-matched peers evident by the high total body fat percentile based on the NHANES [13], in addition to the significant weight gain after starting corticosteroid. Furthermore, the mean TBF percentile did not show a statistically significant change over the study period despite decreasing the mean BMI percentile. This finding raises the concern that some of the reduction in the BMI percentile could reflect a drop in the lean body mass from muscle wasting rather than actual fat loss.

This study has some limitations, including lack of baseline DXA scan;47% of the subjects received treatment before getting referred to our center, and the lack of quantitation of the extent of their physical activity and adipokine measurement. Despite these limitations, the study sheds light on the risk of childhood adiposity after chronic corticosteroid exposure. We hope this study will inspire more research in this area and eventually lead to more intervention-based research to prevent weight gain and excess body fat gain when treating patients with autoimmune diseases.

## Conclusion

Children with Juvenile Dermatomyositis develop significant weight gain in the first year of therapy, evident by the sharp increase in the BMI percentile. Although the BMI percentile decreased throughout the study after the initial spike, the TBF percentile remained high until the end of the study (60 months).

## Supplementary Information

**Additional file 1: Fig. 1s**: Changes of BMI percentile in JDM patients over study duration (60 months). T0 represented baseline date (before initiation of treatment), note data available for around 53% of the study subject. Other time point are T1 > 1.5 years, T2 = 1.51–3.49 years, and T3 = 3.5–5 years. The BMI percentile was calculated based on CDC published charts (https://www.cdc.gov/growthcharts/clinical_charts.htm). Overweight was defined as a BMI at or above the 85th percentile and below the 95th percentile for age and gender-matched children. Obesity was defined as a BMI at or above the 95th percentile for age and gender-matched children and divided into two groups based on severity.

**Additional file 2: Fig. 2s**: Changes of Total body fat (TBF) percentile in JDM patients over study duration (60 months). T1 > 1.5 years, T2 = 1.51–3.49 years, and T3 = 3.5–5 years.

**Additional file 3: Fig. 3s**: Correlation between Total body fat (TBF) percentile at the last visit (T3) and BMI percentile at T1. There was a positive correlation between T1 BMI, and T3 TBF percentile (correlation coefficients 0.63, *P* < 0.001).

**Additional file 4: Fig. 4s**: Correlation between Total body fat (TBF) percentile at the last visit (T3) and initial muscle DAS. There was a positive correlation between TBF percentile at T3 and initial muscle DAS (correlation coefficients 0.49, *P* = 0.001).

## Data Availability

3 supplement figures

## Abbreviations

GC: Glucocorticoids
JDM: Juvenile Dermatomyositis
BMI: Body Mass Index
TBF: Total body fat
DXA: Dual-energy X-ray absorptiometry
CDC: Centers for Disease Control and Prevention
NHANES: National Health and Nutrition Examination Survey
DAS: Disease Activity Score
IBM SPSS: International Business Machines Corporation Statistical Package for the Social Sciences
