# Supplementary figures and images for: Changes in total body fat and body mass index among children with juvenile dermatomyositis treated with high-dose glucocorticoids
Source: Pediatr Rheumatol Online J. 2021 Aug 10;19:118. doi: 10.1186/s12969-021-00622-1 (PMC8353815; doi:10.1186/s12969-021-00622-1)

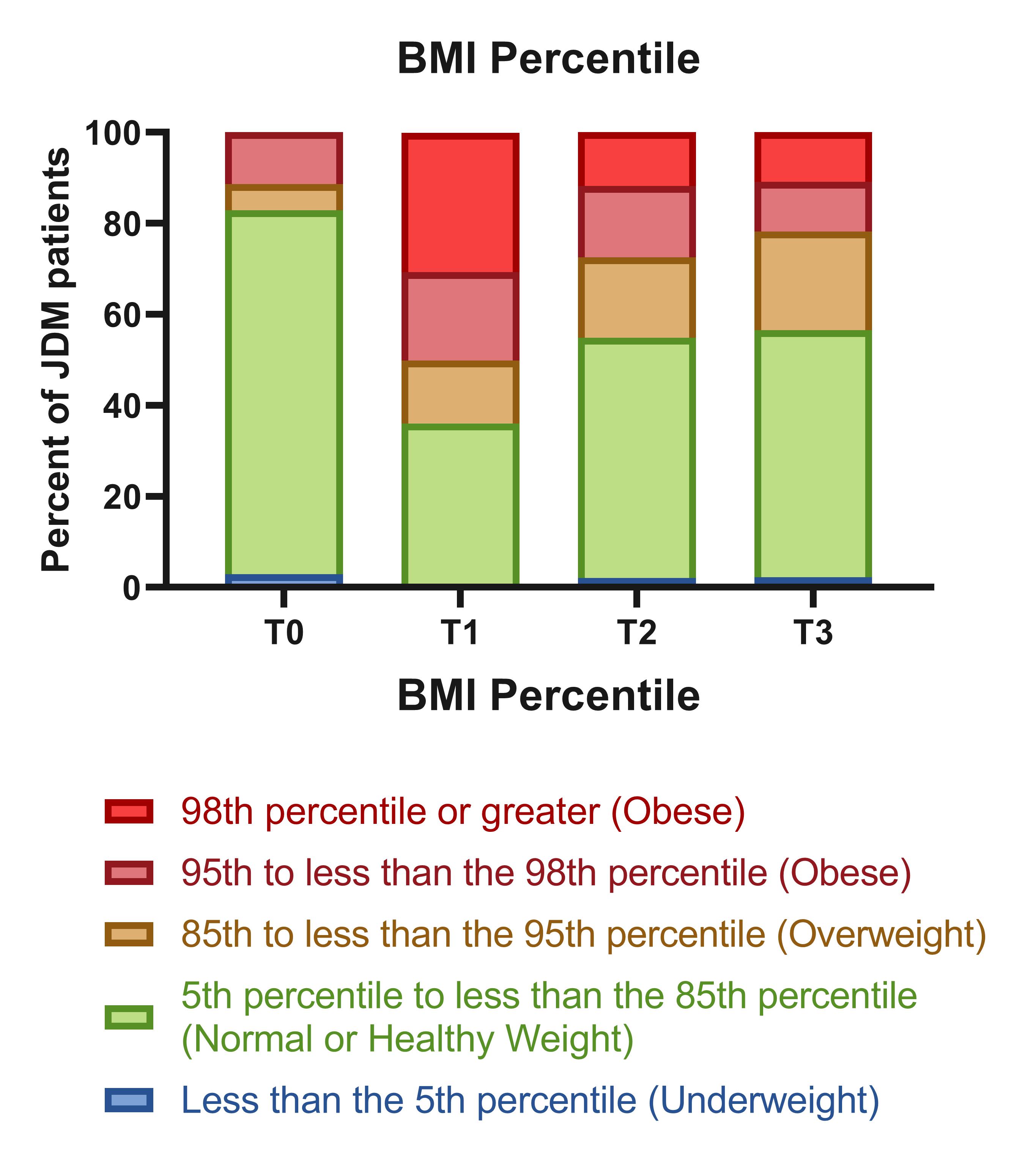

Supplement: Supplementary file 1 — Additional file 1: Fig. 1s: Changes of BMI percentile in JDM patients over study duration (60 months). T0 represented baseline date (before initiation of treatment), note data available for around 53% of the study subject. Other time point are T1 > 1.5 years, T2 = 1.51–3.49 years, and T3 = 3.5–5 years. The BMI percentile was calculated based on CDC published charts (https://www.cdc.gov/growthcharts/clinical_charts.htm). Overweight was defined as a BMI at or above the 85th percentile and below the 95th percentile for age and gender-matched children. Obesity was defined as a BMI at or above the 95th percentile for age and gender-matched children and divided into two groups based on severity. [file 12969_2021_622_MOESM1_ESM.jpg]

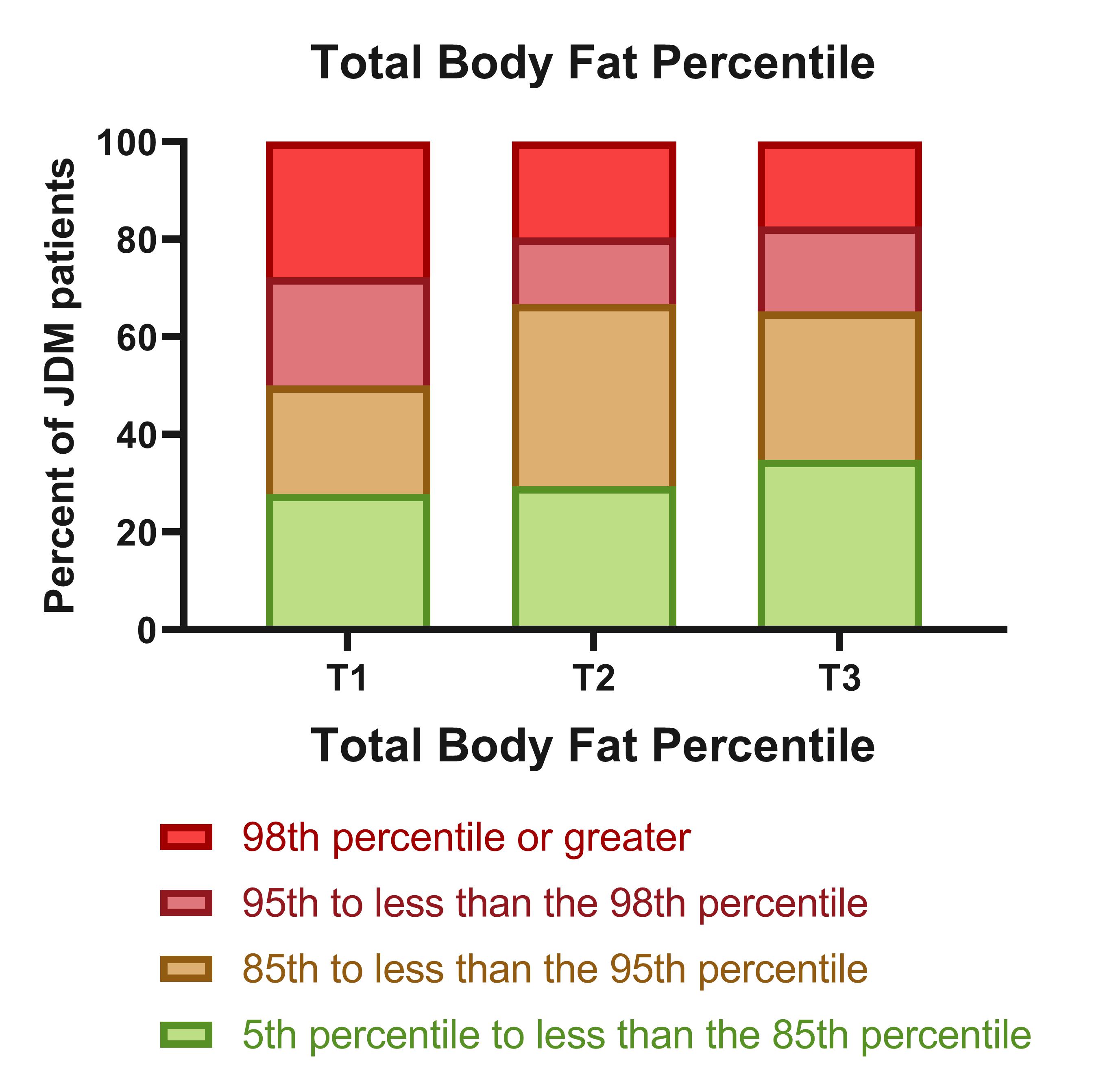

Supplement: Supplementary file 2 — Additional file 2: Fig. 2s: Changes of Total body fat (TBF) percentile in JDM patients over study duration (60 months). T1 > 1.5 years, T2 = 1.51–3.49 years, and T3 = 3.5–5 years. [file 12969_2021_622_MOESM2_ESM.jpg]

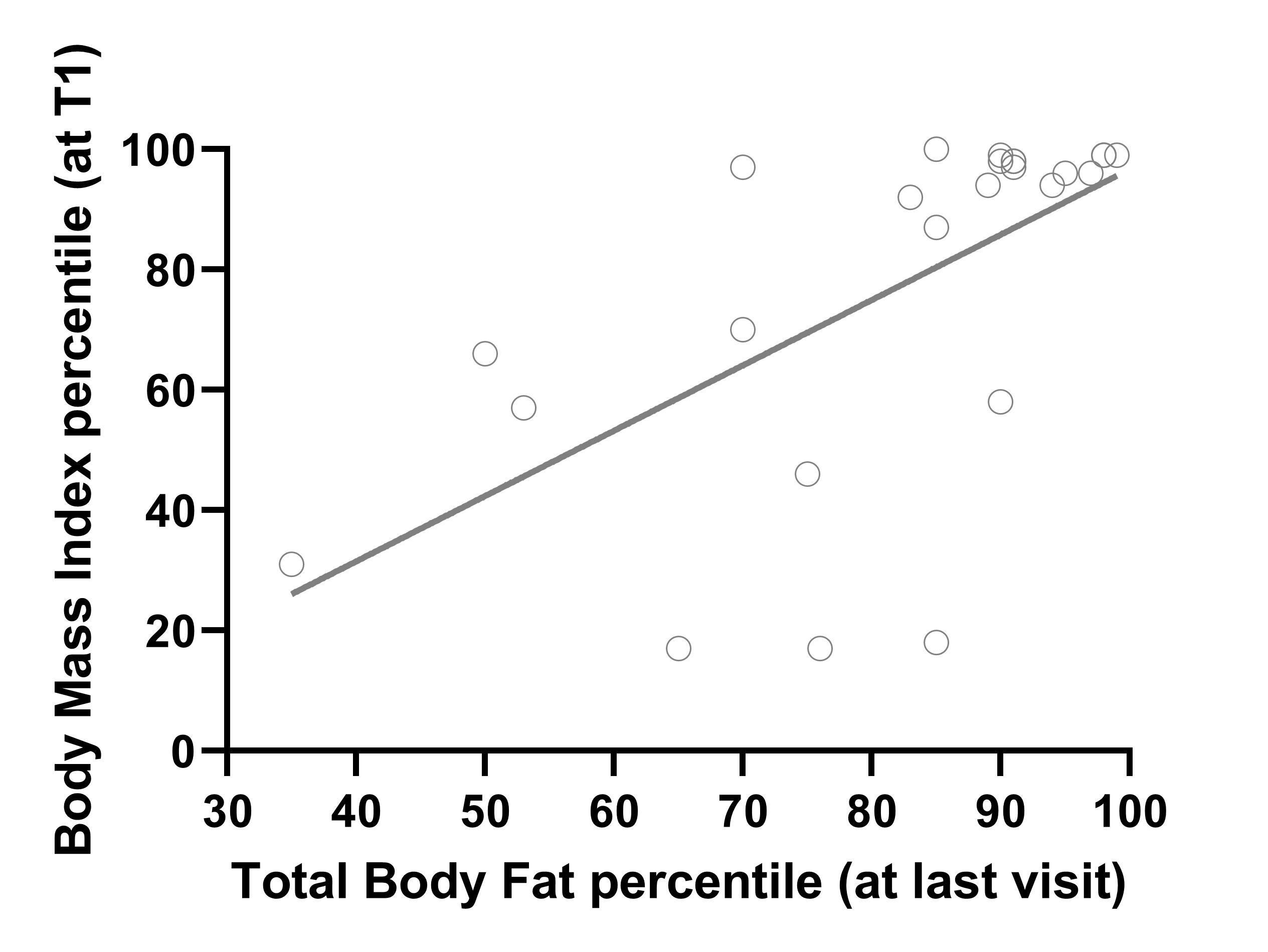

Supplement: Supplementary file 3 — Additional file 3: Fig. 3s: Correlation between Total body fat (TBF) percentile at the last visit (T3) and BMI percentile at T1. There was a positive correlation between T1 BMI, and T3 TBF percentile (correlation coefficients 0.63, P < 0.001). [file 12969_2021_622_MOESM3_ESM.jpg]

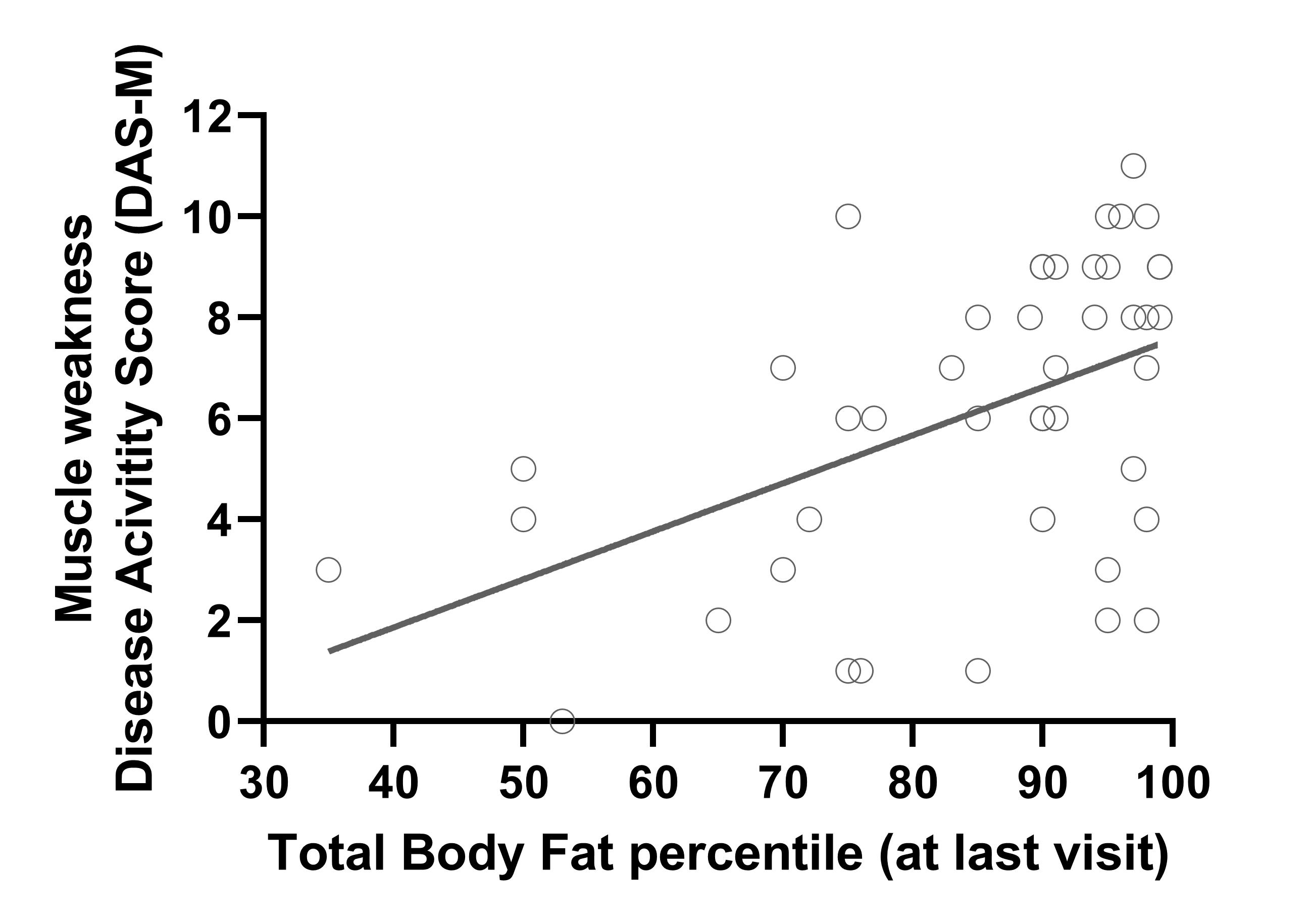

Supplement: Supplementary file 4 — Additional file 4: Fig. 4s: Correlation between Total body fat (TBF) percentile at the last visit (T3) and initial muscle DAS. There was a positive correlation between TBF percentile at T3 and initial muscle DAS (correlation coefficients 0.49, P = 0.001). [file 12969_2021_622_MOESM4_ESM.jpg]
